# Production of xanthan gum from soybean biodiesel: a preliminary study

**DOI:** 10.1186/1753-6561-8-S4-P174

**Published:** 2014-10-01

**Authors:** Davi da S Ferreira, Larissa A de Souza Costa, Márcio Inomata Campos, Mozart D Bispo, Laiza C Krause, Maria Lucila Hernández Macedo, Jorge A Lopez

**Affiliations:** 1 Curso de Farmácia, Universidade Tiradentes, Aracaju, SE, Brazil; 2 Departamento de Engenharia Química, Escola Politécnica, Universidade Federal da Bahia, Salvador, BA, Brazil; 3 Programa de Pós-Graduação em Biotecnologia Industrial, Universidade Tiradentes/Instituto de Tecnologia e Pesquisa, Aracaju, SE, Brazil

## Background

Xanthan gum, a commercial microbial polysaccharide, has many industrial applications, including the tertiary recovery of oil, due to its unique rheological behavior (*e.g*. high viscosity at low concentrations, pseudoplasticity, solubility, stability over a wide range of pH values and temperatures, compatibility with many salts) [1]. Its production employs glucose or sucrose, which raises the price of xanthan production. One way to reduce the cost is to use cheaper alternative substrates, like residues [2]. Biodiesel is included in this context because its chemical composition is susceptible to oxidation, which decreases its capacity as a fuel, resulting in the possibility of organic residue accumulation [3]. Thus, biodiesel conversion into xanthan gum by a fermentation process is one alternative for reducing costs, since the substrate is a critical aspect in its commercial production, and also for minimizing possible environmental impacts. Accordingly, the goal this study was to evaluate the effect of soybean biodiesel as an alternative substrate for non-food grade xanthan gum biosynthesis.

## Methods

Experiments were carried out in a medium containing 2% soybean biodiesel as a carbon source, supplemented with 0.01% urea and 0.1% KH_2_PO_4_, using sucrose as control carbon source under the same operational conditions. In order to obtain the polysaccharide, *Xanthomonas campestris *pv. *campestris *629 was inoculated in these culture media and incubated at 28ºC, 200 min^-1 ^for 96 h. All assays were performed in triplicate. The recovered biopolymer was dried for analysis. The apparent viscosities of the gum solutions from biodiesel and sucrose were measured in a concentric cylinder rheometer coupled to a wash bath for temperature control (25, 45, 65 and 85ºC), with at a shear rate of 25 to 1000 s^-1^. Rheological data were measured according to the Ostwald-de-Waele model (*μ *= *K (γ)^n-1^*), using a regression analysis to ascertain the apparent viscosity (*K, n, R^2^*) [4]. The xanthan FTIR analysis was obtained in the range 4.000-400 cm^-1 ^in KBr pellets at room temperature.

## Results and conclusions

The results showed that soybean biodiesel as an alternative carbon source supported xanthan production with a yield of 12.89 ± 0.61 g×L^-1^. This biopolymer exhibited a consistency index (*K*) of 937.3 ± 0.2 mPa.s and flow rate (*n*) of 0.61 ± 0.01, and R^2 ^= 0.99. These values were similar to those determined for sucrose, the usual carbon source employed for xanthan gum production. The apparent viscosity of xanthan from biodiesel presented a similar profile when compared to gum from sucrose. Regarding the FTIR analysis, both gums showed a similar spectral behavior, probably owing to the polymer structural proximities. The results indicated that biodiesel is a potential and promising option as an alternative substrate for the production of non-food grade xanthan, due to the observed rheological properties indicated through to *K *and *n *values [1,5]. However, fermentation conditions need to be optimized in order to improve yield in this polymer production.
